# On the Vegetable Origin of Diamonds

**Published:** 1865-09

**Authors:** 


### ON THE VEGETABLE ORIGIN OF DIAMONDS.

We have already mentioned that Professor Goeppert obtained the prize offered by the Dutch Scientific Society for an Essay on the vegetable origin of Diamonds, and we are now able to give a short abstract of this highly interesting essay.

Since Lavoisier showed that diamonds were composed of pure carbon, very different opinions have been entertained about their origin, some believing them to be produced by Plutonic, others by Neptunian agency. Newton inclined towards the latter view, and Brewster agrees with him. In 1842 Liebig pronounced the formation of diamonds to be the result of an uninterrupted process of chemical decomposition. "Imagine this chemical decomposition taking place in a fluid rich in carbon, out of which will issne, as a final result of its chemical decomposition, pure carbon, and that in a crystallized form." Indeed, a high temperatnre is adverse to the formation of diamonds, as diamonds become black when subjected to a high degree of temperature, and, according to Despretz's experiments, they are even converted into graphite and coke. The black diamonds, or so-called "carbonates of Bahia," are in part a mixture of uncrystallized carbon and diamonds, as shown by the process of combustion, to which, at my desire, they were submitted by Professor Lowig. That diamonds originated under Neptunian agency is further proved by the frequent occurrence of crystals in them. I have seen them in hundreds of different specimens, and even small cavities containing them. In my essay I have given ample proof that at one time diamonds were soft bodies. Hitherto only one diamond, in the possession of the Emperor of the Brazils, has been known, on which the impression of a grain of sand was visible. I have before me a rhombic dodecahedron, on the whole surface of which impressions of grains of sand are visible, and a similar crystal of the black diamond on which the same impressions exist. In a third there is a cavity with bent and broken crystals of an unknown kind. Two others, an octahedron and a rhombic dodecahedron, have on their surface deep impressions of crystals which are not those of diamonds. The Neptunian origin of diamonds can therefore no longer be doubted. G. Bischof also thinks that, after the discovery of iron pyrites in the diamond, any doubt respecting the formation of diamonds in a moist way has been dispelled. In close connection with these observations is the question about the vegetable origin of diamonds, which in a measure was answered by Newton, who regarded them, on account of their great power of reflecting light, long before their true chemical condition was ascertained, to be coagulated fatty or oily bodies. Jameson and Wilson endeavored to prove this theoretically, Petzholdt practically, by the vegetable cells found in the ashes of diamonds. The vegetable origin of coal and anthracite, and their sedimentary formation, having been thoroughly established, I examined, starting from this point, graphite (hitherto regarded as being without structure, but doubtless having a Neptunian origin) and the diamond; and by the experience I have gained from observing, for a number of years, chalcedony and amber, I am able to distinguish sufficiently between mechanical formations and formations of a vegetable origin. I have not yet attained any results with respect to graphite, but in diamonds I have found numerous foreign bodies enclosed, of which, if they cannot be said to be evidently and undoubtedly vegetable in their origin, it would on the other hand be difficult to deny their vegetable nature altogether. The careful figures which will accompany my essay will enable others to judge on this point, and will, if nothing else, open up the way for further researches.—*London Pharm. Journ.*, July 1, 1865, from *Amer. Journal of Pharmacy.*

